# Time-resolved tracking of the atrioventricular plane displacement in Cardiovascular Magnetic Resonance (CMR) images

**DOI:** 10.1186/s12880-017-0189-5

**Published:** 2017-02-28

**Authors:** Felicia Seemann, Ulrika Pahlm, Katarina Steding-Ehrenborg, Ellen Ostenfeld, David Erlinge, Jean-Luc Dubois-Rande, Svend Eggert Jensen, Dan Atar, Håkan Arheden, Marcus Carlsson, Einar Heiberg

**Affiliations:** 1 Department of Clinical Physiology, Lund University, Skane University Hospital, Lund, Sweden; 2 Department of Numerical Analysis, Faculty of Engineering, Lund University, Lund, Sweden; 3 Department of Biomedical Engineering, Faculty of Engineering, Lund University, Lund, Sweden; 4 Department of Health Sciences, Physiotherapy, Lund University, Lund, Sweden; 5 Department of Cardiology, Clinical Sciences, Lund University, Lund, Sweden; 6 Assistance Publique Hôpitaux de Paris, Hôpital Henri Mondor, Créteil, France; 7 Department of Cardiology, Aalborg University Hospital, Aalborg, Denmark; 8 Department of Cardiology B, Oslo University Hospital Ullevål and Faculty of Medicine, University of Oslo, Oslo, Norway

**Keywords:** Atrioventricular plane displacement, Automated tracking, Normalized cross-correlation, Principal component analysis, Cardiac valve displacement

## Abstract

**Background:**

Atrioventricular plane displacement (AVPD) is an indicator for systolic and diastolic function and accounts for 60% of the left ventricular, and 80% of the right ventricular stroke volume. AVPD is commonly measured clinically in echocardiography as mitral and tricuspid annular plane systolic excursion (MAPSE and TAPSE), but has not been applied widely in cardiovascular magnetic resonance (CMR). To date, there is no robust automatic algorithm available that allows the AVPD to be measured clinically in CMR with input in a single timeframe. This study aimed to develop, validate and provide a method that automatically tracks the left and right ventricular AVPD in CMR images, which can be used in the clinical setting or in applied cardiovascular research in multi-center studies.

**Methods:**

The proposed algorithm is based on template tracking by normalized cross-correlation combined with a priori information by principal component analysis. The AVPD in each timeframe is calculated for the left and right ventricle separately using CMR long-axis cine images of the 2, 3, and 4-chamber views.

The algorithm was developed using a training set (*n* = 40), and validated in a test set (*n* = 113) of healthy subjects, athletes, and patients after ST-elevation myocardial infarction from 10 centers. Validation was done using manual measurements in end diastole and end systole as reference standard. Additionally, AVPD, peak emptying velocity, peak filling velocity, and atrial contraction was validated in 20 subjects, where time-resolved manual measurements were used as reference standard. Inter-observer variability was analyzed in 20 subjects.

**Results:**

In end systole, the difference between the algorithm and the reference standard in the left ventricle was (mean ± SD) -0.6 ± 1.9 mm (R = 0.79), and −0.8 ± 2.1 mm (R = 0.88) in the right ventricle. Inter-observer variability in end systole was −0.6 ± 0.7 mm (R = 0.95), and −0.5 ± 1.4 mm (R = 0.95) for the left and right ventricle, respectively. Validation of peak emptying velocity, peak filling velocity, and atrial contraction yielded lower accuracy than the displacement measures.

**Conclusions:**

The proposed algorithm show good agreement and low bias with the reference standard, and with an agreement in parity with inter-observer variability. Thus, it can be used as an automatic method of tracking and measuring AVPD in CMR.

**Electronic supplementary material:**

The online version of this article (doi:10.1186/s12880-017-0189-5) contains supplementary material, which is available to authorized users.

## Background

The atrioventricular (AV) plane is a fibrous region containing the cardiac valves, separating the atria from the ventricles. During a heartbeat the AV-plane moves as a piston pump, towards apex during systole and back to the initial position during diastole [1, 2]. The atrioventricular plane displacement (AVPD) has been shown to account for 60% of the left ventricular (LV) stroke volume, and 80% of the right ventricular (RV) stroke volume [3, 4] and is an indicator of both systolic and diastolic function [5, 6]. A reduced AVPD is associated with disease and aging [7–10], while regular exercise has been shown to increase AVPD in young male athletes [11] and maintain AVPD at a level similar to young subjects in male master athletes [10].

In echocardiography, it is clinical standard to measure AVPD [9, 12] and a reduced long-axis function has been shown to have prognostic significance for future clinical events [13–17]. Mitral annular plane systolic excursion (MAPSE) is easily used as a good marker for left ventricular function [14], and current guidelines suggest the quantification of tricuspid annular plane systolic excursion (TAPSE) for determining RV function [12, 18]. A number of algorithms has been proposed in order to automatically measure valve displacement with echocardiography [19–21].

In cardiovascular magnetic resonance (CMR) imaging, long-axis cine images, acquired in clinical routine, can be used for measuring AVPD. However, currently AVPD is rarely evaluated in a clinical setting and is not implemented in the consensus document for CMR assessment [22]. When AVPD is assessed in CMR, the required method is often manual measurements in the end diastolic and end systolic timeframe [3, 4, 10, 11, 17, 23–26]. These measurements are applicable for quantification of the maximum displacement over a cardiac cycle. However, it does not carry any information about the temporal distribution of the displacement throughout a heartbeat. Time-resolved measurements allow computation of the AVPD over the whole cardiac cycle, thus creating an AVPD curve. However, manual time-resolved measurements are time consuming and observer dependent. Derived from the AVPD curve, velocity at peak emptying and peak filling can be calculated. These velocities can be considered in parity to the peak systolic and diastolic annular velocity known from echocardiography. Furthermore, atrial contraction can also be derived from the AVPD curve. Automatic tracking of the AVPD reduces the subjectivity of different observers, as well as the time spent on manual image analysis.

A few semi-automatic methods for tracking valves in CMR images have been presented [27–32]. However, no previous method has presented an algorithm using standard clinical CMR images, and that only require manual input in a single timeframe to track the AV-plane in both the left and the right side of heart.

Therefore, the aim of this study was to develop and validate a method that use manually placed input points in a single timeframe and thereafter automatically track the left and right ventricular AV-plane motion in CMR images. This will be useful in the clinical setting as well as in applied cardiovascular research in multi-center studies.

## Methods

### Study population

A total of 153 subjects, who underwent CMR imaging in three previous studies, were included in this study. Subjects consisted of 32 elite athletes [33], 14 elderly normal subjects [10], 81 patients with first time ST-elevation myocardial infarction from the multi-center clinical cardio-protection trial MITOCARE [34], and 26 normal controls [33]. Ten centers participated in data inclusion. Each subject underwent CMR scanning in a 1.5 T scanner using a steady-state-free precession (SSFP) sequence with retrospective ECG gating under end-expiratory breath hold. Scanners were from Philips (Philips Healthcare, Best, The Netherlands), Siemens (Siemens Healthcare, Erlangen, Germany) or GE (GE Healthcare, Waukesha, WI, USA). Long-axis cine images of the 2-chamber, 3-chamber, and 4-chamber views were acquired. In all vendors, typical pixel resolution was 1.5 × 1.5 mm, 8 mm slice thickness, 30 timeframes per cardiac cycle. For Philips, MR sequence parameters ranges were: repetition time 2.6–4.0 ms; echo time 1.3–2.0 ms; flip angle 60°; field of view 151–430 mm; matrix size (92–336) × (92–336). For Siemens ranges were: repetition time 2.6–3.4 ms; echo time 1.1–1.4 ms; flip angle 47–80°; field of view 129–430 mm; matrix size (73–256) × (79–256). For GE: repetition time 3.1–4.0 ms; echo time 1.3–1.7 ms; flip angle 45–70°; field of view 143–400 mm; matrix size (166–512) × (166–512).

Subjects were divided into a training set for algorithm development and optimization (*n* = 40), and a test set for validation (*n* = 113). A subset of the test set (*n* = 20) was used for time-resolved validation while the whole test set was used for validation of the maximum AVPD. The training set consisted of 16 patients, 9 athletes, and 15 healthy controls and was selected manually prior to looking at the images of the included subjects. The time-resolved test set consisted of 7 patients, 6 athletes, 2 elderly normal subjects, and 5 normal controls. Additionally, a subset of *n* = 20 patients from the training set was used for inter-observer variability analysis in end diastole and end systole.

All studies were approved by the local ethical committees, and written informed consent was obtained from each individual.

### Automatic tracking algorithm

**Fig. 1:**
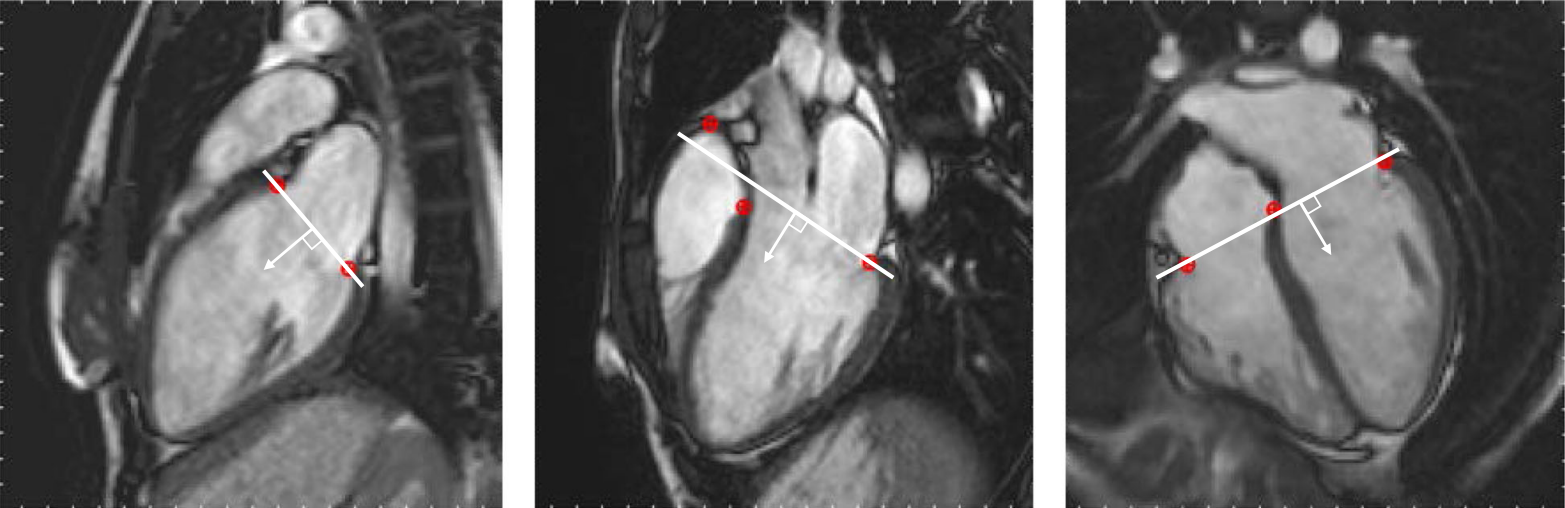
Algorithm initialization and atrioventricular plane definition. The user marks the atrioventricular (AV) plane by placing 8 input points to be tracked, here seen in *red*, in the end diastolic timeframe in the 2-, 3-, and 4-chamber long-axis views. In step 1 of the algorithm, the AV-plane is defined in the end diastolic timeframe of each long-axis view (*white line*), and the direction perpendicular to the AV-plane towards the apex (*white arrow*) marks the direction of the AV-plane displacement (AVPD). In step 2, the a priori position prediction is placed according to the defined plane and direction

The algorithm yield time-resolved curves of the AVPD throughout the cardiac cycle. It was implemented in the medical image analysis software Segment v2.0 R5024 [35] (http://segment.heiberg.se), and is freely available for research purposes. A total of 8 input points that mark the AV-plane are manually placed in the end diastolic timeframe as user input; 2 input points in the 2-chamber view, 3 input points in the 3-chamber, and 3 input points in the 4-chamber. The input points are placed in the most basal part of the compact myocardium in the left and right ventricle, as illustrated in Fig. 1, according to the methodology in the papers by Carlsson et al. [3, 4]. In short, left ventricular AVPD (LVAVPD) is calculated from two input points in each of the three long-axis views for the LV, which equals 6 input points. For the right ventricle, the mean of the two LV septal input points together with the input points of the RV lateral in the 4-chamber view and the RV outflow tract in the 3-chamber view are used for calculating the right ventricular AVPD (RVAVPD). The total LVAVPD and RVAVPD are calculated separately, as described by Carlsson et al. [3, 4]. The algorithm tracks each of the 8 input points separately, using template tracking by normalized cross-correlation [36] combined with a priori information by principal component analysis (PCA) [37].

### Training of statistical model

The algorithm was developed and optimized using the time-resolved manual measurements in the training set (*n* = 40). The amount of timeframes in CMR images differs between the subjects. Hence, all measurements were interpolated to 30 timeframes and normalized by the curve amplitude. For each of the 8 input points, the data was stored as a matrix representation of size 40x30. Rows consisted of the input point displacement perpendicular to the AV-plane in mm for 40 subjects, columns represented the 30 timeframes. Principal component analysis is a method that find underlying structures of the data it is applied to, using statistics and eigenvalue decomposition. Each eigenvector represents a component of the underlying structures, a principal component, and have a corresponding eigenvalue that represent the variance of the structure. In PCA, the largest eigenvalue corresponds to the most dominant principal component of the data. By performing PCA decomposition on the constructed matrix with AVPD data, a statistical model describing the shape of an AVPD curve was constructed. This transforms the matrix into linearly uncorrelated principal components, enabling the information about AVPD from the training set to be represented in 30 dimensions. When analyzing the 30 eigenvalues yielded by the PCA, the sum of the 5 largest eigenvalues accounted for 99% of the sum of all eigenvalues. Hence, the AVPD curve shape and behavior could be reconstructed from 5 coefficient dimensions, using the eigenvectors corresponding to the 5 most significant eigenvalues of the data covariance matrix.

The mean displacement for each point was reconstructed using this model, in order to define a position prediction curve. The prediction points are used by the algorithm as an initial guess of where the input point tracked is located in each timeframe. Reconstruction is also applied on the algorithm tracking result, as a physiological filtering method that smooth the displacement of each tracked point.

**Fig. 2:**
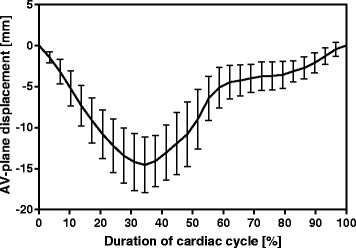
Span of prediction curve. The *solid black line* shows the prediction curve of the septum point in the 4-chamber view, obtained as the mean of the manually measured atrioventricular plane displacement (AVPD) of all subjects in the training set (*n* = 40). The bars marks ± 2SD of 10.000 randomly generated AVPD curves using the five most significant principal component analysis (PCA) eigenvectors multiplied with weights within ± 2SD of the weights calculated from manual measurements, illustrating the span of the prediction curve

The space spanned by the shape reconstruction for one input point, the septum point in the 4-chamber view, is illustrated in Fig. 2. The space was created by 10.000 randomly generated AVPD curves, using the five first principal component eigenvectors multiplied by PCA weights within ± 2SD of the weights of the prediction curve. The spread of the space is shown as bars of ± 2SD over the mean AVPD curve from manual measurements in the training set. The ± 2SD in Fig. 2 has large spread, since the training set include AVPD measures ranging from a reduced AVPD in patients to an increased AVPD in male athletes.

### Tracking and reconstruction process

**Fig. 3:**
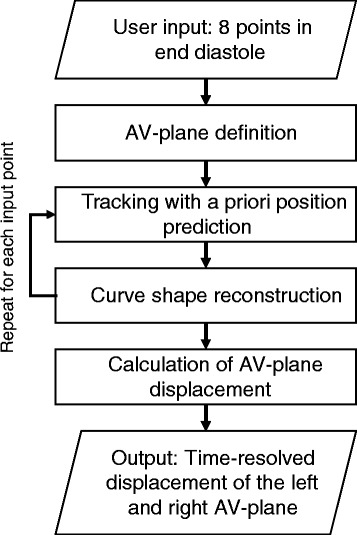
Flow chart of automatic tracking algorithm. The automatic tracking algorithm for the atrioventricular plane displacement (AVPD) tracks 8 input points that are provided as user input throughout the cardiac cycle. The algorithm consists of four processing blocks; definition of the AV-plane, tracking with a priori position prediction, curve shape reconstruction, and calculation of AV-plane displacement and velocity. Processing block 2, in which forward and backward tracking is conducted and merged, and processing block 3, where the curve shape reconstruction is performed, are repeated once for each input point, 8 times. Block 4 is performed once at the end of the algorithm

Figure 3 show a flow chart of the automatic tracking algorithm. The process consists of 4 processing blocks; 1) definition of the AV-plane, 2) template tracking with position prediction, 3) curve shape reconstruction using PCA, and 4) calculation of the AVPD, peak emptying velocity, peak filling velocity, and atrial contraction.

#### Step 1: AV-plane definition

Only the perpendicular movement in relation to the AV-plane in end diastole is accounted for. The AV-plane is defined in each long-axis view. In the 2-chamber and 4-chamber view, the AV-plane is defined as the line where the residuals between the input points and plane are minimized. In the 3-chamber, the plane intersects the lateral input point of the LV and pass between the input points in septum and RV. This is illustrated in Fig. 1.

#### Step 2: tracking with a priori position prediction

The position of the input point throughout the cardiac cycle is predicted by placing prediction points in each timeframe, according to the prediction curves obtained in the training process. Prediction points are positioned perpendicular to the AV-plane, and interpolated according to the duration of the cardiac cycle.

Tracking is performed by extracting the pixels in a region of interest (ROI), a square around the input point in the end diastolic timeframe. In the following timeframe a region of search (ROS) is extracted as a square centered on the prediction point. Normalized cross-correlation is performed for the ROI **I**, indexed as **(x,y)**, and ROS **S**, indexed **(u,v)**. The maximum correlation coefficient between the ROI and ROS, **γ(u,v)**, indicate the best corresponding position of the input point in the second timeframe [36].

$$ \gamma \left( u, v\right)=\frac{{\displaystyle {\sum}_{x, y}}\left[ S\left( x, y\right)-{\overline{S}}_{u, v}\right]\left[ I\left( x- u,\  y- v\right)-\overline{I}\right]}{\sqrt{{\displaystyle {\sum}_{x, y}}{\left[ S\left( x, y\right)-{\overline{S}}_{u, v}\right]}^{2\ }{\displaystyle {\sum}_{x, y}}{\left[ I\left( x- u,\  y- v\right)-\overline{I}\right]}^2}} $$

Around this tracked point, a new ROI is extracted and a ROS is extracted around the prediction point in the next timeframe, and normalized cross-correlation is applied again. The procedure is repeated twice for each input point. First, the tracking is initiated in end diastole, proceeding forward to the next timeframe until reaching the last timeframe. The tracking is then repeated according to the same principle, but instead of forward tracking, the input points in end diastole are tracked backwards.

#### Step 3: curve shape reconstruction

Displacement curves from the forward and backward tracking are calculated and merged as a weighted sum of the curves. The forward tracking displacement is weighted linearly from 1 to 0, assigning the displacement in the first timeframe the weight 1 and the last timeframe the weight 0. Accordingly, the displacement in the first timeframe from backward tracking has the weight 1, and the last timeframe the weight 0. To ensure a smooth and physiologically behaving curve, the merged displacement curve for each point is projected using PCA weights and the 5 most significant eigenvectors.

Each of the 8 input points are tracked separately, hence step 2–3 are repeated 8 times before the fourth processing block is executed. The corresponding position of each input point is visualized in every timeframe in Segment.

#### Step 4: calculation of AV-plane displacement

After tracking, the total LVAVPD and RVAVPD curves are calculated according to Carlsson et al. [3, 4]. The total LVAVPD is the mean displacement curve of the 6 tracked points located in the left side of heart. The sum of the displacement curves of the 2 tracked points in the right side of heart, plus the mean of the displacement curves of the 2 points in septum, divided by 3 gives the total RVAVPD. End systole is defined as the timeframe where the AVPD curve is at its minimum, that is, where the AV-plane is the farthest away from the starting point in end diastole.

**Fig. 4:**
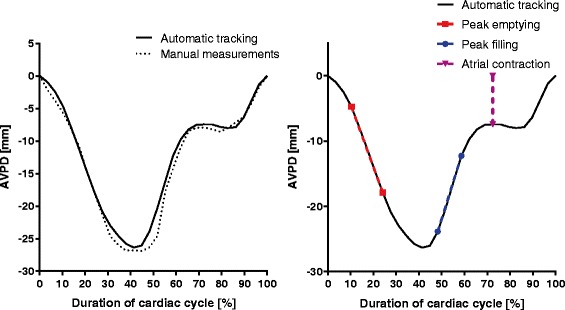
Example of automatic tracking result. A typical result of the atrioventricular plane displacement (AVPD) in the left ventricle (LV) by automatic tracking is shown as a *solid black line* in the left and right panel. A more negative AVPD correspond to a larger displacement. In the *left panel*, the corresponding manually measured AVPD in the LV is shown as a *dotted line*. The *right panel* illustrate how the peak emptying velocity, peak filling velocity, and atrial contraction are obtained. The slope of the *dashed red line* corresponds to the peak emptying velocity, and the slope of the *dashed blue line* corresponds to the peak filling velocity. The length of the *purple dashed line* divided by the AVPD at end systole gives the atrial contraction in %

In order to calculate the velocity at peak emptying and peak filling, the moving average of 3 data samples of the AVPD curve of each side of the heart are calculated, then the first order derivative by forward differentiation is calculated. Peak emptying velocity is calculated as the slope of the line drawn from 2 timeframes before and 2 timeframes after the timeframe where the first order derivative is at its maximum. Peak filling velocity is calculated as the slope of the line from 1 timeframe before and 2 timeframes after the minimum of the first order derivative, see Fig. 4. Atrial contraction is calculated as the distance between the AVPD in end diastole and the AVPD at the timeframe where absolute value of the second derivative of the moving average displacement curve is at its minimum, divided by the AVPD at end systole.

A 3 dimensional (3D) AV-plane was defined for comparison to the 2 dimensional (2D) planes defined in step 1. The 3D plane was defined by the best fit in the least square sense between the input points in all long axis views, using the 3D coordinate system from the MR scanner.

### Parameter optimization

The sizes of the region of interest, ROI, and region of search, ROS, for all 8 input points were optimized by using the time-resolved measurements in the training set as reference. The training set (*n* = 40) consisted of 16 patients, 9 athletes, and 15 healthy controls. The size of each ROI and ROS in mm was optimized over a range of combinations using 10-fold cross-validation. Correlation R value, bias, and standard deviation, SD, was calculated between manual and tracked AVPD in the end systolic timeframe for each parameter combination in an exhaustive search. Also, the 2-norm of the difference between the manual and tracked AVPD curves was calculated for each subject, as a measure of similarity between the manual and automatic AVPD curves, where the value 0 would indicate that the two curves are identical. The ROI and ROS size for each input point was chosen by optimizing the combination of the mean of R value, bias, SD, and 2-norm difference for each fold. The parameter combination yielding the minimum SD was sought out, combined with the requirement that constraints defined for R, bias, and 2-norm were fulfilled. The constraint for R was all parameter combinations yielding an R value above the 75^th^ percentile of all calculated R. For bias, the constraint was parameter combinations yielding a bias below the 25^th^ percentile. The 2-norm constraint was parameter combinations yielding a 2-norm value below the 75^th^ percentile. For 2 out of 8 parameter combinations, the constraints were fulfilled for the global minimum of all calculated standard deviations. The same ROI and ROS sizes are used for the forward and the backward tracking and are presented in Additional file 1.

### Validation

The AV-plane displacement was measured manually by expert readers in all subjects. The automatic tracking algorithm was validated against manual measurements of the total displacement in mm from end diastole to end systole in the whole test set (*n* = 113), as well as separately in the patient (*n* = 65), healthy control (*n* = 24), and athlete (*n* = 24) populations. For the time-resolved subset of the test set (*n* = 20), the AVPD resulting from the automatic tracking algorithm in each timeframe was compared to manual measurements. In the time-resolved test set, the minimum velocity at peak emptying (cm/s), the maximum velocity at peak filling (cm/s), and the atrial contraction (%) was compared for the automatic tracking algorithm and manual measurements. The distance between the manual and automatic AVPD curves was assessed by taking the 2-norm of the difference of the manual and automatic curves in each timeframe. Inter-observer variability of the AVPD in end systole was performed in a subset of 20 patients with first time myocardial infarction.

Since the starting point for the algorithm is the 8 input points provided by the user in the end diastolic timeframe, different input points will result in different tracking results, even if placed only slightly differently. In order to ensure that the same points were compared, the automatic tracking was provided the exact same input points in end diastole as in the manual measurements. To measure how the algorithm results may differ due to different positions of the input points, inter-observer analysis of the algorithm was analyzed. For the inter-observer analysis, both of the algorithm and for manual measurements, the input points in end diastole were placed separately for each observer. All manual measurements were verified by a second observer.

### Statistical analysis

Comparisons were performed using modified Bland-Altman plots with manual measurements as reference standard (mean with limits of agreement (±2SD)) [38], and linear regression analysis (correlation coefficient).

Automatic tracking of the LVAVPD and RVAVPD was compared to manual measurements in end systole for the test set (*n* = 113). In the time-resolved test set (*n* = 20), the displacement in the automatic tracking was compared to time-resolved manual measurements of the displacement in each timeframe. Also, the peak emptying velocity, peak filling velocity, and atrial contraction was compared in the time-resolved test set. Inter-observer variability in end systole was assessed.

## Results

An example of a tracked AVPD curve together with the corresponding manual curve is shown in Fig. 4, showing high similarity of the curves in amplitude and phase. Three movies are available as additional files illustrating typical tracking results in a 2-chamber (Additional file 2), 3-chamber (Additional file 3), and 4-chamber long-axis view (Additional file 4).


Additional file 2:Tracking in a 2-chamber long-axis view.
Additional file 3:Tracking in a 3-chamber long-axis view.
Additional file 4:Tracking in a 4-chamber long-axis view.


**Fig. 5:**
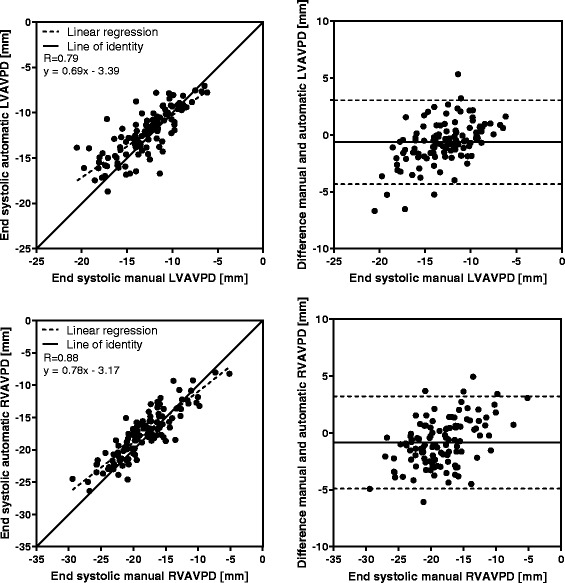
Correlation and bias in the end systolic timeframe for automatic tracking compared to manual measurements. Scatter plot of atrioventricular plane displacement (AVPD) at end systole in mm (*left panel*) and modified Bland-Altman plot of AVPD at end systole in mm (*right panel*) for the automatic tracking against manual measurements in *n* = 113 subjects. Top row shows results for the left ventricle (LVAVPD), and bottom row shows the results for the right ventricle (RVAVPD). The identity line is shown as a *solid line* in the scatter plots, and linear regression as a *dashed line*. A more negative AVPD correspond to a larger displacement. In the Bland-Altman plots the mean bias is shown as a *solid line* with limits of agreement (±2SD) as *dashed lines*. Correlation R value was 0.79 for the left ventricle, and 0.88 for the right ventricle. For the left ventricle, mean bias was −0.6 mm with limits of agreement between −4.3 and 3.1 mm. For the right ventricle, mean bias was −0.8 mm with limits of agreement between −4.9 and 3.3 mm

**Fig. 6:**
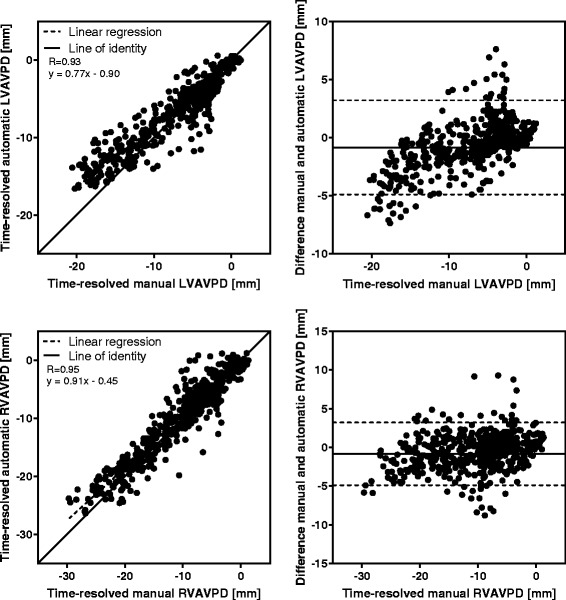
Correlation and bias in all timeframes for automatic tracking compared to manual measurements. Scatter plot (*left panel*) and modified Bland-Altman plot (*right panel*) for automatic tracking against manual measurements of the atrioventricular plane displacement (AVPD) in *n* = 20 subjects in all timeframes. Top row shows results for the left ventricle (LVAVPD), and the bottom row shows the results for the right ventricle (RVAVPD). The identity line is shown as a *solid line* in the scatter plots, and linear regression as a *dashed line*. A more negative AVPD correspond to a larger displacement. In the modified Bland-Altman plots the mean bias is shown as a solid line with limits of agreement (±2SD) as *dashed lines*. Correlation R value was 0.93 for the left ventricle, and 0.95 for the right ventricle. For the left ventricle, mean bias was −0.6 mm with limits of agreement between −4.5 and 3.3 mm. For the right ventricle, mean bias was −0.5 mm with limits of agreement between −4.8 and 3.8 mm

**Fig. 7:**
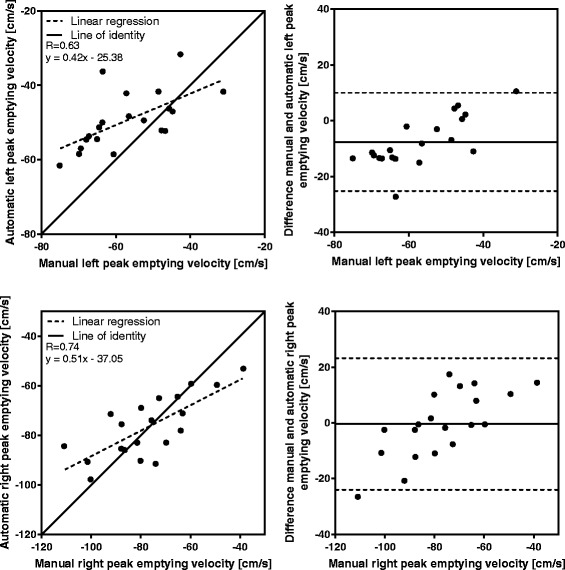
Correlation and bias for the velocity at peak emptying, automatic tracking compared to manual measurements. Scatter plot of the peak emptying velocity in cm/s (*left panel*) and Bland-Altman plot of peak emptying velocity in cm/s (*right panel*) for the automatic tracking against manual measurements in *n* = 20 subjects. Top row shows results for the left ventricle and the bottom row shows the results for the right ventricle. The identity line is shown as a *solid line* in the scatter plots, and linear regression as a *dashed line*. In the Bland-Altman plots the mean bias is shown as a *solid line* with limits of agreement (±2SD) as *dashed lines*. Correlation R value was 0.63 for the left ventricle, and 0.74 for the right ventricle. For the left ventricle, mean bias was −7.6 cm/s with limits of agreement between −25.2 and 10.0 cm/s. For the right ventricle, mean bias was −0.4 cm/s with limits of agreement between −23.9 and 23.1 cm/s

**Fig. 8:**
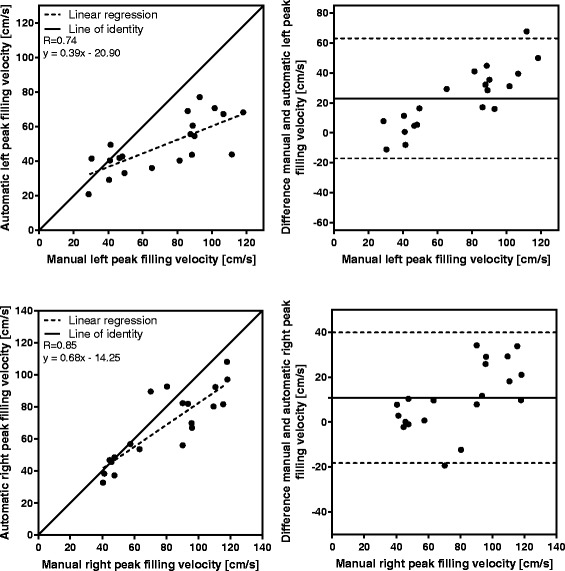
Correlation and bias for the velocity at peak filling, automatic tracking compared to manual measurements. Scatter plot of the peak filling velocity in cm/s (*left panel*) and Bland-Altman plot of peak filling velocity in cm/s (*right panel*) for the automatic tracking against manual measurements in *n* = 20 subjects. Top row shows results for the left ventricle and the bottom row shows the results for the right ventricle. The identity line is shown as a *solid line* in the scatter plots, and linear regression as a *dashed line*. In the Bland-Altman plots the mean bias is shown as a *solid line* with limits of agreement (±2SD) as *dashed lines*. Correlation R value was 0.74 for the left ventricle, and 0.85 for the right ventricle. For the left ventricle, mean bias was 23.0 cm/s with limits of agreement between −17.0 and 63.0 cm/s. For the right ventricle, mean bias was 10.8 cm/s with limits of agreement between −18.2 and 39.8 cm/s

**Fig. 9:**
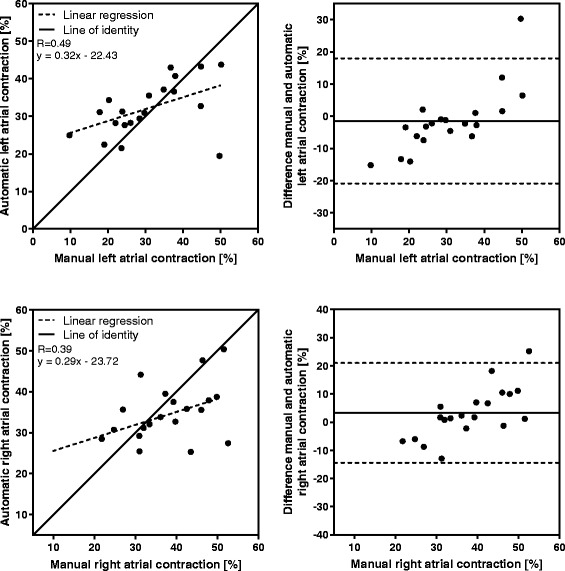
Correlation and bias for the atrial contraction, automatic tracking compared to manual measurements. Scatter plot of the atrial contraction in % of the AVPD in end systole (*left panel*) and the corresponding modified Bland-Altman plot (*right panel*) for the automatic tracking against manual measurements in *n* = 20 subjects. Top row shows results for the left ventricle and the bottom row shows the results for the right ventricle. The identity line is shown as a *solid line* in the scatter plots, and linear regression as a *dashed line*. In the modified Bland-Altman plots the mean bias is shown as a *solid line* with limits of agreement (±2SD) as *dashed lines*. Correlation R value was 0.49 for the left ventricle, and 0.39 for the right ventricle. For the left ventricle, mean bias was −1.5% with limits of agreement between −20.9 and 17.9%. For the right ventricle, mean bias was 3.3% with limits of agreement between −14.3 and 20.9%

Manual measurements compared to automatic tracking results at end systole (*n* = 113) are shown in Fig. 5, and the displacement difference of the time-resolved manual measurements (*n* = 20) compared to automatic tracking in each timeframe are shown in Fig. 6. For both displacement measures, strong correlation and low bias was found between manual and automatic AVPD measurements. The results for the velocity at peak emptying (*n* = 20) for manual compared to automatic measurements are shown in Fig. 7, and the results for the velocity at peak filling (*n* = 20) in Fig. 8. The algorithm tends to overestimate the left peak emptying velocity, and underestimate the left and right peak filling velocity. Figure 9 shows the results for the atrial contraction (*n* = 20).

Manual and automatic measurements of the AV-plane displacement in all subjects (*n* = 113) are presented in Table 1 as correlation R value and bias ± SD, while Table 2 present the population based AVPD results for patients (*n* = 65), healthy controls (*n* = 24), and athletes (*n* = 24). LVAVPD correlation R value was lower in athletes than for healthy subjects and patients. Otherwise, displacement results in the different populations are in parity to those in all subjects. Results for automatically calculated peak emptying velocity, peak filling velocity, and atrial contraction are presented in Table 3. The 2-norm of the difference between the time-resolved manual and tracked AVPD curves was 10 ± 5 for the left side of the heart, and 11 ± 6 for the right side of the heart. The tracking yielded displacement results agreeing with manual measurements, with a low bias and high correlation R value. For the peak emptying velocity, peak filling velocity, and atrial contraction, the results presented a higher bias and SD than for the displacement results.

**Table 1:** Displacement results. Manual and automatic measurement results of atrioventricular plane displacement (AVPD) as mean ± SD in the left (LVAVPD) and right ventricle (RVAVPD). Comparison of automatic tracking vs manual measurements as correlation R value and bias ± SD of the AVPD. In the time-resolved measure, the AVPD in each timeframe is compared for *n* = 20 subjects. The validation in end systole was performed on *n* = 113 subjects

Measure	Manual	Automatic	R	Bias ± SD
LVAVPD in end systole [mm]	−13 ± 3	−12 ± 3	0.79	−0.6 ± 1.9
RVAVPD in end systole [mm]	−18 ± 4	−18 ± 4	0.88	−0.8 ± 2.1
Time-resolved LVAVPD [mm]	-	-	0.93	−0.6 ± 2.0
Time-resolved RVAVPD [mm]	-	-	0.95	−0.5 ± 2.2

**Table 2:** Population based end systolic displacement results. Manual and automatic measurement results of atrioventricular plane displacement (AVPD) in end systole as mean ± SD in the left (LVAVPD) and right ventricle (RVAVPD) for patients (*n* = 65), healthy controls (*n* = 24), and athletes (*n* = 24). Comparison of automatic tracking vs manual measurements as correlation R value and bias ± SD of the AVPD

Measure	Manual	Automatic	R	Bias ± SD
LVAVPD in patients [mm]	−11 ± 2	−11 ± 2	0.81	−0.6 ± 1.3
RVAVPD in patients [mm]	−17 ± 4	−15 ± 3	0.83	−1.0 ± 2.2
LVAVPD in healthy controls [mm]	−14 ± 3	−14 ± 2	0.49	0.0 ± 2.5
RVAVPD in healthy controls [mm]	−20 ± 4	−20 ± 3	0.88	−0.4 ± 1.7
LVAVPD in athletes [mm]	−16 ± 2	−15 ± 2	0.44	−1.3 ± 2.3
RVAVPD in athletes [mm]	−22 ± 3	−21 ± 2	0.77	−0.7 ± 2.2

**Table 3:** Results for automatically calculated peak velocities and atrial contraction. Manual and automatic measurement results of automatically calculated peak emptying velocity, peak filling velocity, and atrial contraction as mean ± SD in the left and right side of the heart. Comparison of automatic tracking vs manual measurements as correlation R value and bias ± SD. The validation was performed on *n* = 20 subjects. The atrial contraction is given in % of the atrioventricular plane displacement (AVPD) in end systole

Measure	Manual	Automatic	R	Bias ± SD
Left peak emptying velocity [cm/s]	−57 ± 12	−49 ± 8	0.63	−7.6 ± 9.0
Right peak emptying velocity [cm/s]	−77 ± 18	−77 ± 12	0.74	−0.4 ± 12.0
Left peak filling velocity [cm/s]	72 ± 29	49 ± 15	0.74	23.0 ± 20.4
Right peak filling velocity [cm/s]	79 ± 28	68 ± 23	0.85	10.8 ± 14.8
Left atrial contraction [%]	31 ± 11	32 ± 7	0.49	−1.5 ± 9.9
Right atrial contraction [%]	38 ± 9	35 ± 7	0.39	3.3 ± 9.0

Inter-observer variability for manual measurements in end systole (*n* = 20) was −0.6 ± 0.7 mm, R = 0.95, for the LVAVPD and −0.5 ± 1.4 mm, R = 0.95, for the RVAVPD. Thus, inter-observer variability was similar to the difference between manual and automatic algorithm measurement of AVPD. Inter-observer variability of the algorithm in end systole (*n* = 20) was 0.2 ± 1.4 mm, R = 0.69, for the LVAVPD and 0.0 ± 0.7 mm, R = 0.98, for the RVAVPD. The average time for tracking and calculation of the AVPD for both the left and the right heart was 0.6 s per subject, using a standard laptop computer.

**Fig. 10:**
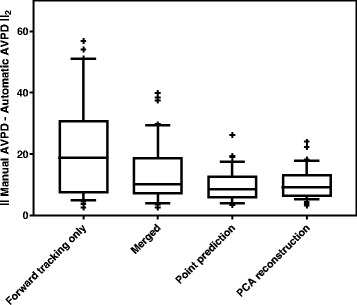
Added value of algorithm processing blocks. Box plot illustrating the added value of performing the tracking both forwards and backwards using point prediction and curve shape reconstruction. Each box contains the results as the 2-norm of the difference between the manually measured atrioventricular plane displacement (AVPD) curve and the tracked AVPD curve, from the time-resolved test set (*n* = 20) in both the left and right side of the heart. In the first box, the 2-norm between the curves when only tracking forward from the end diastolic timeframe to the last timeframe is shown, without any position prediction. Second (Merged), the 2-norm is shown when merging the curves resulting from the forward and backward tracking. Third (Point prediction), the 2-norm is shown when merging the curves from the forward and backward tracking and also using point prediction. And last (PCA reconstruction), the 2-norm when merging with point prediction, and also reconstructing the curve shape using the principal component analysis (PCA) eigenvectors is shown. In each box, the central mark is the median. The box edges represent the 25^th^ and 75^th^ percentiles, and the whiskers indicate the 10^th^ and 90^th^ percentiles. Outliers are represented as plus signs (+)

Figure 10 illustrates the added value, in terms of the 2-norm of the difference between the time-resolved manual and tracked AVPD curves, for four steps in the algorithm; only tracking forward without position prediction, merging the tracking forward and backwards, merging curves using point prediction, and finally when also adding the curve shape reconstruction. Each processing block yielded results with lower variability.

Comparison of tracking results using 2D and 3D AV-planes in the time-resolved test set (*n* = 20) showed a low variability and strong correlation. LVAVPD results was −0.1 ± 0.5 mm, R = 0.99. RVAVPD results was −0.1 ± 0.6 mm, R = 1.00.

## Discussion

In this study, an automatic algorithm for time-resolved tracking of the AVPD from standard long-axis CMR cine images has been developed and validated. The algorithm is based on template tracking by normalized cross-correlation and a priori information by principal component analysis, and yield the position of the AV-plane in all timeframes. The input needed by the user is the marking of the AV-plane in end diastole at eight points. The validation of the algorithm in 113 subjects from multi-center and multi-vendor studies showed low bias and high correlation to expert manual measurements.

Forward template tracking can be quite unstable. If the tracking fails in one timeframe, the error will propagate since another point than the intended will be tracked in the coming timeframes. This might occur at any timeframe for various reasons such as poor image quality, or a region of search (ROS) that does not cover the intended match. This problem has previously been approached in different ways. In the method presented by Wu et al. [29], the user was encouraged to interactively adjust unsatisfactory tracking results, and then run the algorithm again based on the corrections. In the work by Leng et al. [28], stability is obtained by placing input points in several timeframes, and tracking is performed in the timeframes between these input points. In the method presented by Maffessanti et al. [27], manual input is required in the end diastolic and end systolic timeframes. A novelty in the presented algorithm compared to previous approaches is that input points are only required in the end diastolic timeframe and merges the tracking results from both forward and backward tracking in order to approach the stability issues with forward tracking. In case the template tracking drifts off at some point, the weighted sum of the two displacement curves aids in improving the final AVPD curve. Also, the use of a priori information specific to each input point tracked is used as a prediction for the tracking, thus always placing the ROS in a region where the match is expected, which stabilizes the forward tracking. The prediction adapts according to the number of timeframes in the data set, varying heart rates, and the parameter optimization of ROI and ROS sizes in mm allow the algorithm to adapt to different spatial resolutions. The incremental value of merging two tracking results, using position prediction and curve shape reconstruction can be seen in Fig. 10, suggesting that these steps improves the tracking results comparing to only performing forward tracking without any position prediction.

Other researchers have implemented valve tracking for different purposes than to quantify AV-plane displacement per se. Quantification of mitral valve displacement and velocity in 4-chamber view cine images was presented by Saba et al. [30], and a similar method quantifying the tricuspid valve displacement and velocity has been described by Ito et al. [31]. The method by Saba et al. [30] was later on expanded to a three-dimensional volume tracking method, based on semi-automated tracking of the mitral valve in the 2, 3, and 4-chamber views [29]. Also, Westenberg et al. implemented retrospective valve tracking in order to measure three-dimensional blood flow in CMR [32]. The presented algorithm in this study aimed to quantify the AV-plane displacement in both the left and the right side of the heart, using cine images of the 2, 3, and 4-chamber view that are acquired as clinical standard. To the best of our knowledge, this approach of using images acquired as clinical standard to quantify both the left and right ventricular AVPD has not be published before.

Manual inter-observer variability in end systole was performed on 20 subjects and showed a low bias and variability, and manual measurements were used as reference standard. The inter-observer variability of the algorithm in end systole had a similar agreement as for the manual inter-observer variability in the right side of the heart. In the left side of the heart, the algorithm inter-observer had a lower correlation R value than for the manual inter-observer, but low bias and SD. Hence, the overall algorithm performance varies about as much as manual measurements vary for different observers. Also, the displacement results of the algorithm had an agreement in parity with the manual inter-observer variability, and bias was below pixel resolution for both automatic and manual displacement measurements. The end systolic results for both the LVAVPD and the RVAVPD were interchangeable with the inter-observer variability. In the three population groups (consisting of patients, healthy controls, and athletes), similar displacement results were obtained, although the correlation R value was stronger in the LVAVPD for patients compared to healthy controls and athletes.

The AV-plane determined in 2D, by defining a line in each long axis view, was compared to an approach where the AV-plane was defined in 3D, by a best fit all input points in a least square sense. The comparison showed a strong correlation, low bias and variability between both methods. Images were acquired during end-expiratory breath hold and the apex is essentially stationary during the heart beat [39], hence the global heart movement was minimized during the data collection. However, in case the subject move during the MR scan, the 3D coordinate system from the MR scanner might not correspond well to the obtained long axis image positions. Therefore, the 2D implementation was used in this study.

Peak emptying and peak filling are usually calculated from ventricular volume curves and reported as a volumetric flow rate in ml/s. In this study the velocity of the AV-plane at peak emptying and peak filling are quantified in cm/s. Since the AVPD accounts for 60% of the stroke volume in the left side of the heart and 80% in the right side [4], the AV-plane velocity could be an indirect measure of the emptying and filling of the ventricles. A study by Leng et al. showed a good correlation between early diastolic filling from tissue Doppler echocardiography and CMR measures of the AV-plane velocity which would support this [28]. The derivative of a distance curve must be calculated in order to determine velocity estimations. The tracking yields a time-resolved AVPD curve, and even though the curve shape is reconstructed and smoothed using PCA eigenvectors, this curve can appear noisy in between the timeframes. Also, difficulties are to be expected since CMR cine images can be rather noisy in the timeframes where the peak emptying velocity and peak filling velocity occur. Therefore, taking the numerical first order derivative of the AVPD curve results in an even noisier velocity curve, where it is hard to extract an accurate maximum and minimum velocity. Hence, taking the moving average of the displacement curves prior to differentiating them, and calculating the slope of the straight line over the timeframes where the maximum and minimum velocity occur was considered a more stable approach in this study. The results for the peak emptying velocity have an agreement for manual and automatic measurements in the left and the right side of the heart. When comparing the results of the peak filling velocity, the automatic measurements tends to underestimate the velocities and both bias and SD are quite large.

In general, it is difficult to automatically determine the atrial contraction in the AVPD curve, since the duration of the diastasis can vary from about 20% of the cardiac cycle for low heart rates, to non-existing for high heart rates [40]. The proposed algorithm is programmed to calculate atrial contraction to be the difference in AVPD between end diastole and where the absolute value of the second derivative of the AVPD curve is at its minimum, but the user might change the interval by observing the tracked curve. By design, the algorithm forces the AVPD curve to return to its original position at the end of the cardiac cycle. Hence, if the AVPD tracking is far away from the original position at diastasis, the atrial contraction will be represented as a very steep segment in the AVPD curve. This might explain why the SDs of the atrial contraction was large. Also, the diastasis can be hard to detect for high heart rates.

An underestimation can be seen for large displacements in both the end systolic and time-resolved AVPD measurements (Figs. 5 and 6), as well as in the results of high peak velocities and large atrial contractions (Figs. 7, 8 and 9). The cause of this underestimation is unknown. However, one source for the underestimation could be an effect of overtraining of the algorithm. In order to evaluate if overtraining could be a cause, the proportion of subjects in the training and test sets were compared. The proportion of healthy controls in the training set was 38% whereas in the test set it was 21%, thus the training set included more subjects with a higher AVPD compared to the test set. The proportion of athletes in both the training and test set were the same (23% and 21%, respectively). This should rather be a source for overestimation than underestimation. Another potential source of error might be that the algorithm has difficulties in tracking the AV-plane when it is moving at high velocities. On the other hand, the overall tracked displacement curve has a good agreement to manual measurements, according to the time-resolved displacement validation and the low distance between the automatic and manual AVPD curves, measured by the 2-norm.

### Limitations

Limitations of this study comprises that patients included in the training and test set were only patients with first time ST-elevation myocardial infarction. The algorithm is based on a priori information extracted from manual measurements in the training set. Hence other patient categories and anatomic variations may have been missed when constructing the AVPD prediction model, such as patients with very low AVPD. Including such patients might be necessary for the algorithm to track very low AV-plane motions, but it might also reduce the accuracy of the tracking results in healthy subjects if the prediction curve is reduced in amplitude. On the other hand, the lower limit of agreement of LVAVPD was 7 mm, why this limitation might be of less significance.

The algorithm is not expected to work on prospective ECG gated CMR data, since the backward tracking, PCA prediction, and curve shape reconstruction are designed on data comprising the whole cardiac cycle. All images in this study were acquired during end-expiratory breath hold. In images acquired during breathing, the calculation of the AVPD might be obfuscated by the chest movement.

The input points to be tracked are chosen by the user, and variations in placements of these input points may result in some points being hard to track for the algorithm. Heart sizes impacts the amplitude of the AVPD curve, where for example athletes have larger hearts and therefore are expected to have a larger AVPD, while elderly and different patient groups often have a reduced AVPD. The amplitude of the tracking predictor is not scaled according to the specific subject, and even though the size of the ROS’s for each input point has been optimized for a large range of AVPD amplitudes, the true match may not be present in the ROS for varying heart sizes. The curve shape reconstruction ensures that the AV-plane trajectory is transformed to a smooth and physiological movement. If the chosen input point does not move as physiologically expected, for example due to anatomical variations, severe disease, or an incorrect input by the user, the calculated displacement may not represent the true AVPD. Therefore, manual monitoring and manual corrections are important for research and clinical use, as for any automatic image analysis algorithm. The same applies to the estimated peak emptying velocity, peak filling velocity, and atrial contraction, where the user should check that the interval of the lines defining the measures are placed in agreement with the appearance of the AVPD curve. The use of the proposed automatic tracking algorithm will reduce time input and user variability, which are important aspects for clinical use. Also, this allow the researcher or clinician to observe not only the total AVPD in end systole, which is the commonly used reference standard today, but also the whole AVPD trajectory throughout the whole cardiac cycle.

### Future work

Atrial contraction results were not satisfactory, comprising many outliers (Fig. 9). Further studies of the algorithm can verify if this is a recurring issue.

In order to implement a fully automatized algorithm for AVPD tracking, the 8 manually placed input points in the end diastolic timeframe may possibly be replaced by automatic input point detection. The tracking algorithm predict the position of each input point in the next timeframe, using a priori information gathered from normal controls, athletes and patients with first time myocardial infarction. Gathering information from other physiological variations and patient groups might improve the prediction model. An automatic adaptive approach to scale the prediction curve based on the physiology of each individual data set could also increase the robustness of the algorithm and yield more accurate results for displacement, peak velocities, and atrial contraction.

## Conclusion

The developed automatic algorithm performs time-resolved tracking of the AVDP in CMR images using normalized cross-correlation and a priori information based on principal component analysis. The algorithm performs well with regards to displacement against manual measurements in healthy controls of a wide age spread, athletes, and first time myocardial infarction patients from multi-center and multi-vendor data. Further, the algorithm yields displacement results in parity with inter-observer variability.

Therefore, an algorithm based on normalized cross-correlation and principal component analysis may be introduced as a method for measuring the atrioventricular plane displacement in CMR imaging.

## Additional file

Additional file 1: Results of parameter optimization. (PDF 354 kb)

## Abbreviations

AV: Atrioventricular
AVPD: Atrioventricular plane displacement
CMR: Cardiovascular magnetic resonance
LV: Left ventricle
LVAVPD: Left ventricular atrioventricular plane displacement
MAPSE: Mitral annular plane systolic excursion
PCA: Principal component analysis
ROI: Region of interest
ROS: Region of search
RV: Right ventricle
RVAVPD: Right ventricular atrioventricular plane displacement
SD: Standard deviations
TAPSE: Tricuspid annular plane systolic excursion
