# Supplementary material for: Time-resolved tracking of the atrioventricular plane displacement in Cardiovascular Magnetic Resonance (CMR) images
Source: BMC Med Imaging. 2017 Feb 28;17:19. doi: 10.1186/s12880-017-0189-5 (PMC5330030; doi:10.1186/s12880-017-0189-5)
Supplement: Additional file 1: — Results of parameter optimization. (PDF 354 kb) [file 12880_2017_189_MOESM1_ESM.pdf]

Results of parameter optimization

2 chamber 3 chamber 4 chamber

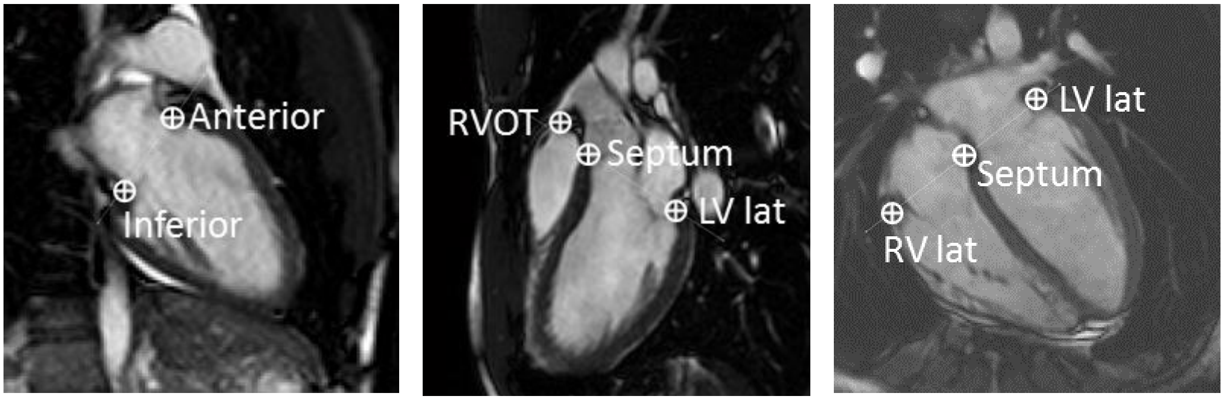

Schematic drawing and naming of points in each long-axis view used by the algorithm.

Long-axis view	Point	ROI size [mm]	ROS size [mm]
2 chamber	Inferior	5	9
2 chamber	Anterior	7	12
3 chamber	Septum	9	17
3 chamber	LV lat	7	26
3 chamber	RVOT	7	23
4 chamber	Septum	5	11
4 chamber	LV lat	10	12
4 chamber	RV lat	7	13

Optimization results for the length of the side of the region of interest (ROI) and region of search (ROS) squares in mm, according to long-axis views and point naming in the figure above.
